# An Unusual Case of Tongue Entrapped in an Electronic Nail Clipper: A Case Report

**DOI:** 10.7759/cureus.60744

**Published:** 2024-05-21

**Authors:** Bader Fatani, Nouf A Alshehri, Haya A Alsagr, Mohammed Alkindi

**Affiliations:** 1 Dentistry, College of Dentistry, King Saud University, Riyadh, SAU; 2 Oral and Maxillofacial Surgery, King Saud University, Riyadh, SAU

**Keywords:** nail clipper, laceration, entrapment, foreign object, tongue

## Abstract

Children with entrapped foreign objects in their tongues require a careful and knowledgeable approach to calm their anxiety, as not all patients may be appropriate for moderate sedation. Deciding the best treatment approach is often challenging due to conflicting advice and unclear guidelines. Recently, the emergency department has started favoring natural healing over suturing, especially for small tongue lacerations not involving the tip of the tongue. However, in cases of large lacerations or involvement of the tip of the tongue, suturing is usually recommended. This case report presents a rare incident of a tongue trapped in an electronic nail device in a pediatric patient.

## Introduction

The common types of self-harm involve cutting, burning, scratching, bruising, biting, and disrupting wound healing. The body areas most often affected are the head with the surrounding tissues, the hands, and the neck [1]. Tongue lacerations are common in children, often resulting from falls or sports injuries. Challenges are often faced in deciding the best treatment due to conflicting advice and a lack of clear guidelines. It's uncertain for some physicians which lacerations should be sutured, and which could heal on their own. Recently, the pediatric emergency department has moved towards recommending secondary wound healing over suturing, especially for tongue lacerations less than 2 cm long without involving the tip, as suggested by Seiler et al.'s study [2]. Moreover, some authors suggested that suturing tongue lacerations in young children do not lead to better outcomes or post-injury recovery [3]. In this case report we demonstrate a rare case of an entrapped tongue in an electronic nail device which was managed accordingly to release the entrapped tongue.

## Case presentation

**Figure 1:**
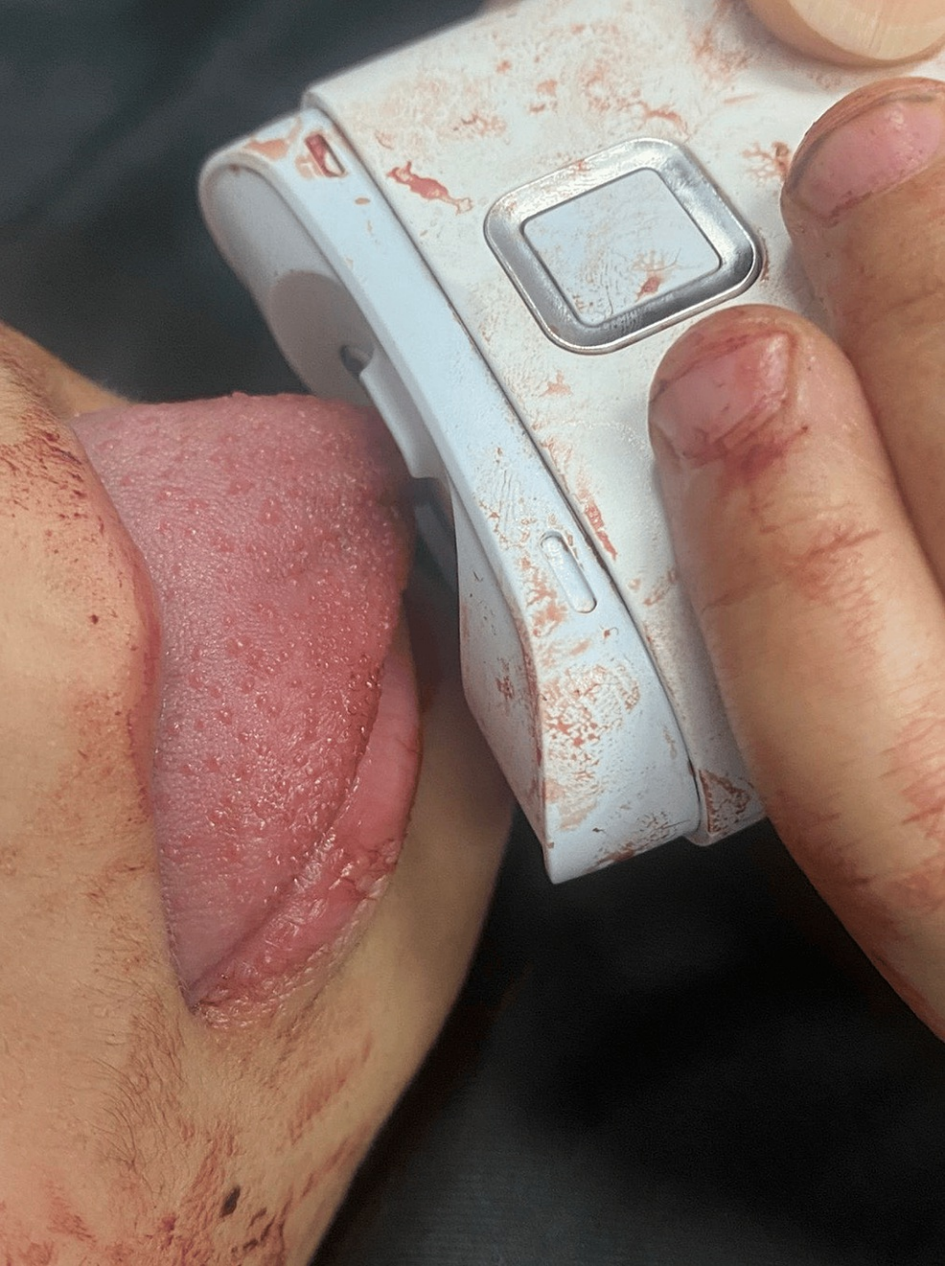
Tongue entrapped in an electronic nail clipper.

A 4-year-old male patient with a non-relevant medical history came to the emergency department with his parents after sustaining sudden entrapment of the child’s tongue into a working electronic-powered nail clipper. The parents described the incidents as a sudden entrapment of the child’s tongue to the nail clipper while the child was playing with the device and accidentally turning the device on which pulled the tongue tip into the circulating device blade. The child was screaming in pain while arriving at the emergency department while the parents reported multiple failed attempts to remove the device which resulted in further bleeding and pain for the child. Demonstration of the entrapped tongue in Figure 1. 

**Figure 2:**
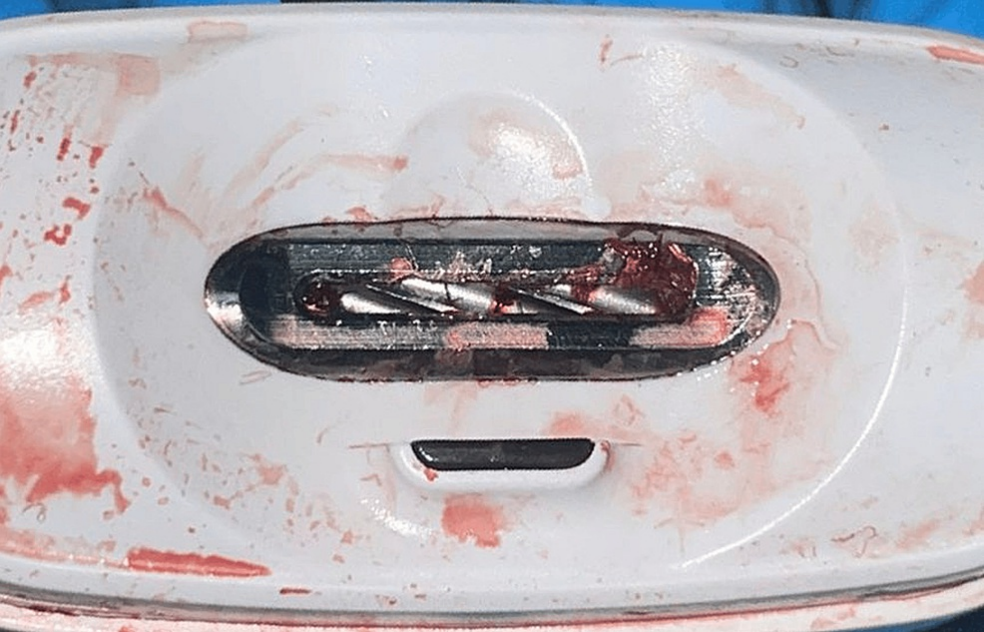
Magnified blade shape of the electronic nail clipper.

The patient was conscious and extremely anxious with negative behavior and sudden movements due to severe pain and anxiety. The patient was monitored primarily by the emergency team with pain medications and behavioral management methods. After the oral and maxillofacial team arrived, a full assessment of tongue status as well as the device and blade shape were evaluated to determine the proper release of the tongue avoiding further tongue injury. Figure 2 shows a magnified image of the blade shape.

**Figure 3:**
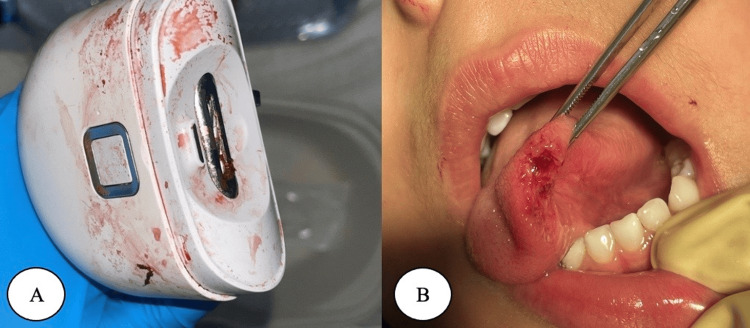
A. Showing the nail clipper after the removal. B. Showing the tongue after the separation.

The patient was monitored primarily, and the treatment was decided to release the entrapped tongue using a small skin hook to push the tongue outside the blade. The plan was to start with the local anesthetic instead of conscious sedation because the airway could not be maintained due to device obstruction. Moreover, in case of oxygen desaturation during sedation, an oxygen mask will not be able to be placed as the device will interfere with an oxygen mask. The patient was given a 2% lidocaine as an infiltration on the tip of the tongue. The device was accessed from the side to try to push the tongue outside avoiding the risk of mistakenly turning on the device. After multiple attempts, the tongue was released from the device. Figure 3 shows the device and the tongue lacerations following the release. 

**Figure 4:**
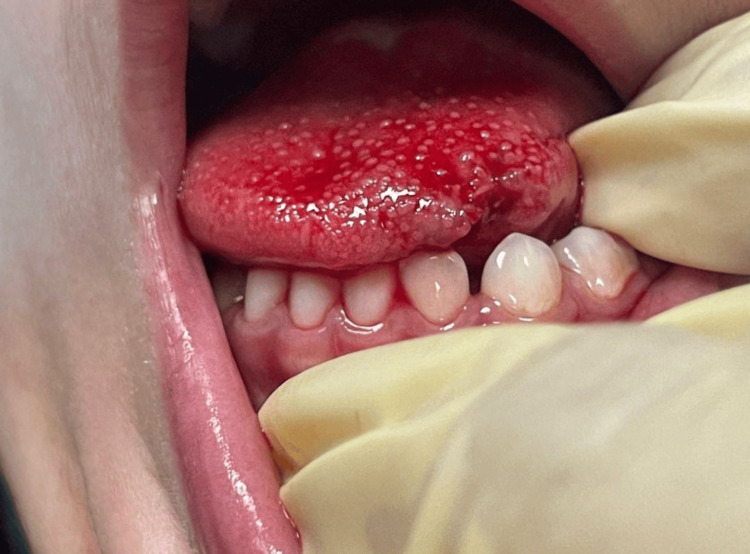
Showing the tongue tip after suturing of the lacerated area.

After the removal of the device, the patient was anesthetized using propofol as a conscious sedation. The tongue laceration was then sutured using Vicryl 4-0 in Figure 4. The patient was sent home with oral analgesic with no further complications encountered in the follow-up visits.

## Discussion

Before attempting the removal of an entrapped foreign body from the mouth, it is essential to understand the anatomy of that specific area. The tongue, a muscular organ, relies on both intrinsic and extrinsic muscles for movement. The primary blood supply to the tongue comes from the lingual artery, a branch of the external carotid artery, which runs close to the midline underneath the tongue. The lingual artery gives off branches such as the dorsal lingual, suprahyoid, and sublingual arteries, which support the tongue's robust collateral circulation and tissue regeneration potential. Veins of the tongue follow the arteries closely and drain into the internal jugular vein. Sensory and motor functions of the tongue are controlled by branches of cranial nerves V, VII, and IX [4]. Guha et al reported a case involving a previously healthy 10-year-old girl who was brought to the emergency department with her tongue stuck tightly inside a 9 oz glass bottle of chocolate drink. The tongue was swollen and bruised due to restricted blood flow caused by the bottle's neck. After unsuccessful attempts to free the tongue, a glass expert was called to surgically remove the bottle using a steel glass cutter in the operating room. A small blood clot was also drained with a needle to relieve pressure on the tongue, leading to quick improvement [5]. 

Shah et al paper explored tongue entrapment in an aluminum water bottle, covering removal techniques and airway care. They also examined sedated mechanical removal of the trapped object and transnasal fiberoptic intubation [6]. Guo et al study focused on recognizing characteristics that could aid in diagnosing foreign objects remaining on the tongue. They suggested considering the presence of a foreign body in cases of persistent wounds or tongue enlargement, especially when a radiopaque line appears on CT scans regardless of patients having a history of foreign body ingestion. Enhanced detection of tongue foreign bodies is achievable through specific CT imaging techniques like thin-slice reconstruction and detailed analysis [7]. 

Yee et al reported on a case involving a 4-year-old girl who arrived at the emergency department with her tongue trapped in a specially designed plastic bottle. Despite trying gentle methods like pulling, breaking the vacuum seal, and using lubricants, the foreign object could only be removed by cutting the rigid plastic into two pieces using a water-cooled high-speed dental tool [8]. Baloda et al described a scenario where a 5-year-old boy came to the emergency room with a wooden block stuck in his mouth following a fall. The block was stuck behind his primary central incisors without causing visible mouth or dental damage. Initial attempts to remove it failed due to the child’s nervousness and muscle spasms. To help with anxiety and muscle relaxation, the patient received intravenous diazepam, and a tenaculum was used to remove the object [9]. Due to the infrequent occurrence of these cases, the treatment often necessitates an inventive and creative strategy to safely remove the object without worsening or harming the patient [10].

## Conclusions

This report discusses an unusual case of tongue entrapped in an electronic nail clipper that was managed by accessing the device thoroughly in order to remove the detained tongue. Children with foreign objects entrapped in their mouths need a cautious approach and knowledge to reduce patients’ anxiety, as some patients may not be suitable for moderate sedation. Emergency physicians should be aware of possible issues like palatal injury, temporomandibular joint dislocation, epiglottitis, and retained foreign objects. In cases of tongue entrapment, the tongue is often dragged from the entrapped object. However, securing the airway should be a priority. In case of bleeding, regular suctioning is necessary. The emergency department should have the proper tools for the removal process, these include medic shears, Dykes, and an orthodontic wire cutter for precise cutting.
